# Diseases of the Spinal Cord

**Published:** 1898-06

**Authors:** 


**Diseases of the Spinal Cord.** By Byrom Bramwell, M.D. Third Edition. Pp. xii., 659. Edinburgh: William F. Clay. 1895.

It is hardly necessary to review at length the third edition of a book so widely and so favourably known as Dr. Byrom Bramwell's *Diseases of the Spinal Cord,*—one, too, that has been translated into three languages. As was inevitable in view of the growth of our knowledge with regard to nervous diseases, a considerable extension of the book has been made in this edition. It appears to us to have been brought thoroughly to date, and the abundance and excellence of the illustrations, more particularly the clinical ones, deserve special mention. Such clinical pictures of disease are especially valuable to the student. The leading characteristic of the work is its eminently practical character, and the author's experience in, and aptitude for, teaching enables him to put his subject-matter in a particularly clear and attractive form, so that the student and practitioner will find the book a useful guide. In this edition the text is arranged in the form of lectures. We have no doubt that it will meet with the same favour that has been accorded to the former editions. It is only right to call attention to the clearness of the type in which the book is printed.

